# Effect of climate on surgical site infections and anticipated increases in the United States

**DOI:** 10.1038/s41598-022-24255-w

**Published:** 2022-11-16

**Authors:** Raymond J. Liou, Michelle J. Earley, Joseph D. Forrester

**Affiliations:** 1 School of Medicine, Stanford University, Stanford, USA; 2 Division of General Surgery, Department of Surgery, Stanford University, 300 Pasteur Drive, H3638, Stanford, CA 94305 USA

**Keywords:** Medical research, Diseases, Infectious diseases, Public health

## Abstract

Surgical site infections (SSI) are one of the most common and costly hospital-acquired infections in the United States. Meteorological variables such as temperature, humidity, and precipitation may represent a neglected group of risk factors for SSI. Using a national private insurance database, we collected admission and follow-up records for National Healthcare Safety Network-monitored surgical procedures and associated climate conditions from 2007 to 2014. We found that every 10 cm increase of maximum daily precipitation resulted in a 1.09 odds increase in SSI after discharge, while every g/kg unit increase in specific humidity resulted in a 1.03 odds increase in SSI risk after discharge. We identified the Southeast region of the United States at highest risk of climate change-related SSI, with an estimated 3% increase in SSI by 2060 under high emission assumptions. Our results describe the effect of climate on SSI and the potential burden of climate-change related SSI in the United States.

## Introduction

Surgical site infections (SSI) cause morbidity, mortality, and excess healthcare expenditure among patients undergoing surgery. They are one of the most common hospital-acquired infections (HAI) in the United States, with an estimated 13,100–158,000 SSIs per year following inpatient surgery^1,2^. Patients who develop SSI are twice as likely to die and on average stay 10 days longer in the hospital^3,4^. SSIs increase each patient’s medical bill by at least $20,000, and are one of the most costly infections in the United States with an estimated annual expenditure of $5.5 billion^2^. Due to the heavy burden SSIs place on patients and the healthcare system, much effort has been devoted to identifying SSI risk factors and developing mitigation strategies. However, current research and prevention strategies focus predominantly on perioperative and patient characteristics—the impact of meteorological variables such as temperature, humidity, and precipitation are less well understood^5–9^.

Effects of climate on the distribution of many infectious diseases are well documented. Precipitation and temperature have been shown to increase spread of water and vector-borne diseases, while soft tissue infection and antibiotic-resistant microbes have also been shown to increase with temperature^10–16^. As the planet’s climate continues to change, burden of these diseases is expected to rise^17^. Yet data describing relationships between SSIs and meteorological variables remains sparse^9^. Studies using single-center and national healthcare datasets have shown a temporal relationship between specific surgical procedures and SSI occurrence, with peak SSI rates occurring during summer months^18–22^. However, relationships between specific climate conditions such as temperature, humidity, and precipitation and SSI are not well-studied and these studies do not analyze the effect of regional variability on climate-related SSIs^9^.

Our objective was to evaluate effects of temperature, humidity, and precipitation on SSI rates in the United States across a range of surgical procedures using a high-spatial, high-temporal resolution national patient dataset. We describe the effect of meteorological variables and seasonality on SSI after adjusting for patient and procedural risk factors, stratify regions and surgical procedure categories by their climate-related SSI risk, and predict burden of SSI due to climate change across various future scenarios.

## Methods

### Epidemiological data acquisition

We obtained procedure and SSI occurrence data from the IBM^®^ MarketScan^®^ Research Databases^23^. This dataset includes 6 de-identified claims databases capturing retrospective patient-specific information on healthcare expenditures, inpatient and outpatient claims data, outpatient prescription claims, and clinical utilization records, comprising 273 million individual patients in the United States^24^. MarketScan^®^ claims data has previously been used to analyze health outcomes, drug utilization, and cost-analysis for a variety of surgical procedures^25–27^. For the assessment of SSI rates, we extracted inpatient and outpatient records from the MarketScan^®^ Commercial and Medicare Supplemental Databases for the years 2007–2014. As no identifiable data was extracted this study was determined to be exempt by the Stanford University Institutional Review Board.

Patient records were filtered using the NHSN operative code list of procedures monitored for SSI occurrence^28^. These codes represent 39 general procedure categories and include common procedures in general surgery, obstetrics, vascular surgery, cardiothoracic surgery, neurosurgery, orthopedic surgery, transplant surgery, and urology^29^. The pacemaker group was omitted from analysis. International Classification of Diseases (ICD)-10 Procedure Coding System (PCS) codes were converted to ICD-9 Clinical Modification (CM) using the 2018 General Equivalence Mapping, with 1:1 and approximate matches manually reviewed for inclusion. The final list of ICD-9 CM and Current Procedure Terminology^®^ (CPT) procedure codes were used to filter patient records for inclusion in the study (Supplemental Table 1).

Each NHSN procedure category group has an SSI surveillance window of either 30 or 90 days^30^**.** For each patient record where an NHSN surgical encounter occurred, all follow-up records occurring within the designated surveillance window were queried. Relevant variables extracted included admission or encounter date, diagnostic and procedure codes, year, admission status, length of stay, age-adjusted Charlson Comorbidity Index, sex, Medicare status, and metropolitan statistical area (MSA) wherein the encounter occurred. SSI was defined by presence of ICD9 codes 998.51 and 998.59 in either admission or follow-up records.

Duplicate records, patients ages < 18 or > 109 years old, records with missing information, surgical encounters occurring outside the continental US, qualified surgical encounters that occurred within 90 days of another surgical procedure, and surgical encounters with procedures belonging to two or more surgical procedure categories were excluded except in cases where small bowel occurred with colon surgery and when ovarian procedures occurred with hysterectomy.

### Climate data acquisition

Climate data was extracted from the Gridded Surface Meteorological (gridMET) dataset, a surface meteorological dataset of the United States from 1979 to the present^31^. GridMET data was accessed via Google Earth Engine, a cloud-based geospatial processing platform^32^. MSA boundaries for climate extraction were derived from U.S. Census TIGER/Line shapefiles for the years 2007–2014^33^. Weather data gathered for each MSA included daily minimum temperature (°C), daily maximum temperature (°C), specific humidity (kg/kg), and precipitation (mm/day) between 2007 and 2014. We calculated average and maximum (as well as minimum in the case of temperature) values of these variables across moving 30- and 90-day windows. Recorded procedures were matched to appropriate weather windows with day of admission occurring during the 15th day of the weather window. We used a 15-day lead time in order to account for possible effects of weather conditions on microbial skin colonization prior to procedure occurrence.

### Predictors and confounders

We selected known SSI risk factors for inclusion in the data analysis such as age-adjusted Charlson Comorbidity Index scores, sex, length of stay, surgery procedure category, MSA, and Medicare status^34–37^. In January 2012, a new mandate required hospitals to report their SSIs to the NHSN, which the Centers for Medicare and Medicaid Services used to qualify hospitals for their annual payment update^38^. We included year of procedure to account for coding changes potentially associated with this policy transition. Age was not included as an independentpredictor as CCI already accounts for age. NHSN category groups were organized into 15 general procedure categories to reduce model complexity (Table 1).

**Table 1:** Newly created categories for multivariate multinomial analysis using existing NHSN procedure category groups for SSI monitoring. The NHSN pacemaker group was omitted from analysis. AVSD: Shunt for dialysis.

New categories (n = 15)	NHSN categories (n = 38)
Caesarean section	Caesarean section
Hernia	Hernia
Abdominal	Gallbladder, colon, appendectomy, gastric, biliary, small bowel, rectal, spleen, exploratory laparotomy
Breast	Breast
Prosthesis	Knee prosthesis, hip prosthesis
Fracture	Fracture
Spine	Laminectomy, fusion
Gynecology	Ovary, hysterectomy, vaginal hysterectomy
Thoracic	Thoracic, cardiac, coronary artery bypass
Neuro	Cranial, ventricular shunt
Neck	Thyroid, neck
Amputation	Amputation
Urology	Prostate, kidney
Vascular	AVSD, peripheral bypass, abdominal aortic aneurysm, carotid endarterectomy
Transplant	Kidney transplant, liver transplant, heart transplant

### Statistical analysis

Univariable and multivariable multinomial logistic regression models were employed to evaluate the association between climate variables and SSI. These analyses were performed in STATA (StataCorp, Release 16, College Station, TX)^39^. For univariable multinomial models, all weather variables were analyzed independently, with outcome levels: no SSI; SSI reported during surgical encounter; and SSI reported in follow-up during surveillance window period. To address issues of extremely large or small odds ratios, units of specific humidity and precipitation were converted to g/kg from kg/kg and cm/day from mm/day, respectively. Only one covariate for precipitation, specific humidity, and temperature was included in the multivariable multinomial model, with selection discrimination involving effect size comparisons between different variables, literature evidence of the effect of the measured variable and SSI occurrence, and a significance cutoff of p < 0.1. The final multinomial fixed effects model included the chosen climate covariates from univariable analyses and the aforementioned predictors and confounders. P-values were adjusted using Holm’s correction. Designed for astrophysics applications, the Lomb-Scargle periodogram is a spectral analysis method similar to a Fourier transform, with added benefit of computing statistics for periodogram peaks^40,41^. To determine seasonality of SSI occurrence we calculated SSI rates for every month between 2007 and 2014 and used the “lomb” package in R (R Foundation for Statistical Computing, version 3.5.1, Vienna) to detect any significant periodicity^42^. Once periodicity was determined, we calculated time of maximum SSI occurrence by fitting the previously defined non-linear equation:

1 $$\left({y}_{i},{t}_{i}\right)=(a{t}_{i}+b)\bigg(1+\epsilon cos\bigg(\frac{{t}_{i}-\phi }{T}\bigg)\bigg)$$

where *a* and *b* define the linear trend, ϵ strength of seasonality, and φ phase and month of highest SSI occurrence^11^.

### Regional susceptibility analysis

To determine areas at risk for future climate-associated SSI, we obtained predicted precipitation, humidity, and temperature across two Representative Concentration Pathways (RCPs) for 2040 and 2060 using coupled atmosphere–ocean general circulation models (AOGCMs) from the Coupled Model Intercomparison Project 5 (CMIP5). The two RCPs chosen were RCP 4.5 and RCP 8.5, with the former representing a medium stabilization emission scenario and the latter representing a high emission scenario^43^. Temperature and precipitation were obtained from the NASA Earth Exchange Downscaled Climate Projections, which includes downscaled projections from 33 AOGCMs^44^. Surface specific humidity was gathered from 11 AOGCMs (Supplemental Table 2). Using the multivariate multinomial climate coefficients, odds ratios for climate-related SSI from future scenarios compared to 2010 were calculated for each MSA.

## Results

### Descriptive statistics

In total, 7,702,846 records from 393 MSAs met inclusion criteria, including 4,303,447 (55.9%) inpatient and 3,399,399 (44.1%) outpatient records (Table 2). SSI incidence across all procedures was 1.6%, with 18.6% diagnosed during the procedure admission and 81.4% diagnosed during follow-up. Patients with female sex underwent 5,175,830 (67.2%) procedures. Patients with Medicare supplemental insurance paid by employers represented 1,456,559 (18.9%) records, with the remaining on private employer-sponsored insurance. Mean (SD) length of stay across all procedures was 3.5 (5.3) days.

**Table 2:** Descriptive statistics of qualified MarketScan^®^ procedures records (n = 7,702,846). SSI: Surgical site infection.

Variable	All		No SSI		SSI during admission		SSI after discharge	
	Mean (SD)	Range	Mean (SD)	Range	Mean (SD)	Range	Mean (SD)	Range
Age (years)	50.2 (16.8)	18–108	50.2 (16.8)	18–108	55.3 (14.9)	18–98	50.1 (16.3)	18–105
Charlson comorbidity Index	2.4 (2.9)	0–26	2.4 (2.9)	0–26	3.9 (3.4)	0–22	2.8 (3.1)	0–23
Length of stay (days)	3.5 (5.3)	1–541	3.5 (5.2)	1–541	10.2 (11.9)	1–307	4.5 (6.8)	1–342
Maximum daily precipitation (mm/day)	33.6 (21.2)	0–231	33.6 (21.2)	0–231	32.1 (20.8)	0–221	33.9 (21.4)	0–221
Mean daily precipitation (mm/day)	2.9 (1.8)	0–21	2.9 (1.8)	0–21	2.9 (1.9)	0–16.6	2.9 (1.8)	0–18.5
Maximum daily specific humidity (kg/kg)	0.012 (0.004)	0.001–0.02	0.012 (0.004)	0.001–0.02	0.011 (0.004)	0.001–0.02	0.011 (0.004)	0.002–0.02
Mean daily specific humidity (kg/kg)	0.007 (0.004)	0.001–0.02	0.007 (0.004)	0.001–0.02	0.007 (0.004)	0.001–0.02	0.007 (0.004)	0.001–0.02
Maximum daily maximum temperature (°C)	28.7 (7.6)	− 4.3–47.7	28.7 (7.9)	− 4.3–47.7	28.1 (8.1)	0.2–46.3	28.9 (7.8)	− 2.2–47.7
Mean daily maximum temperature (°C)	19.7 (9.8)	− 12.9–43.2	19.7 (9.8)	− 12.9–43.2	19.5 (9.9)	− 10.6–42.3	20 (9.7)	− 11.8–43.2
Minimum daily maximum temperature (°C)	9.4 (11.7)	− 27.1–39.9	9.4 (11.7)	− 27.1–39.9	9.8 (11.7)	− 25–38.5	9.6 (11.6)	− 26.5–39.9
Maximum daily minimum temperature (°C)	16 (7.1)	− 16.7–31.7	16 (7.1)	− 16.7–31.7	15.6 (7.3)	− 11.2–31.6	16.2 (7.1)	− 13.7–31.4
Mean daily minimum temperature (°C)	7.7 (8.8)	− 27.2–28.6	7.7 (8.8)	− 27.2–28.6	7.6 (8.9)	− 21.9–27.3	8 (8.8)	− 24.1–27.9
Minimum daily minimum temperature (°C)	− 1.3 (10.6)	− 38.2–25.5	− 1.3 (10.6)	− 38.2–25.5	− .94 (10.7)	− 37.6–24.1	− 1 (10.6)	-38.2–25.1

The abdominal procedure category group accounted for the largest portion of procedures (n = 1,635,175, 21.2%), followed by the caesarean section procedure group (n = 1,008,056, 13.1%), prosthesis procedure group (n = 870,623, 11.3%), and gynecology group (n = 814,686, 10.6%) (Supplemental Table 3). The procedure category groups with fewest procedures were the transplant (n = 9361, 0.12%), amputation (n = 132,616, 1.7%), and vascular (n = 147,146, 1.9%) procedure groups.

### Univariable analysis

Results of univariable multinomial logistic regression models with climate variables are described in Table 3. Across precipitation covariates, maximum daily precipitation (cm/day) was the most significant predictor for SSI during admission (OR: 0.97, 95% CI 0.96–0.97), and SSI after discharge (OR: 1.007, 95% CI 1.004–1.01). Across specific humidity covariates, maximum daily specific humidity (g/kg) was the only variable significantly associated with both SSI during admission (OR: 0.99, CI 0.99–0.99) and SSI after discharge (OR: 1.01, 95% CI 1.01–1.01), whereas mean daily specific humidity was only significant in the model for post-discharge SSI. Thus, maximum daily precipitation and maximum daily specific humidity were included in the multivariable multinomial model.

**Table 3:** Univariable multinomial analysis coefficients and odds ratios for climate predictors. Specific humidity and precipitation units were converted to g/kg from kg/kg and cm/day from mm/day, respectively, to reduce exponential power of odds ratios. Selection for inclusion in the multivariable analysis took into consideration variable effect size, significance, and literature support for inclusion.

	SSI during admission			SSI after discharge		
	B (SE)	OR (95% CI)	p	B (SE)	OR (95% CI)	p
Maximum daily precipitation (cm/day)	− 0.034 (0.003)	0.97 (0.96–0.97)	< 0.001	0.007 (0.001)	1.007 (1.004–1.01)	< 0.001
Mean daily precipitation (cm/day)	0.065 (0.035)	1.07 (1.00–1.14)	0.06	0.049 (0.017)	1.05 (1.02–1.09)	0.004
Maximum daily specific humidity (g/kg)	− 0.01 (0.002)	0.99 (0.99–0.99)	< 0.001	0.008 (0.007)	1.01 (1.01–1.01)	< 0.001
ean daily specific humidity (g/kg)	− 0.0007 (0.0016)	1.00 (1.00–1.00)	0.65	0.008 (0.0008)	1.01 (1.01–1.01)	< 0.001
Maximum daily maximum temperature (°C)	− 0.009 (0.0008)	0.99 (0.99–0.99)	< 0.001	0.004 (0.0004)	1.004 (1.003–1.004)	< 0.001
Mean daily maximum temperature (°C)	− 0.002 (0.0007)	0.998 (0.996–0.999)	0.004	0.003 (0.0003)	1.003 (1.002–1.003)	< 0.001
Minimum daily maximum temperature (°C)	0.003 (0.0005)	1.003 (1.002–1.004)	< 0.001	0.002 (0.0003)	1.002 (1.001–1.002)	< 0.001
Maximum daily minimum temperature (°C)	− 0.008 (0.0009)	0.99 (0.99–0.99)	< 0.001	0.005 (0.0004)	1.005 (1.004–1.005)	< 0.001
Mean daily minimum temperature (°C)	− 0.001 (0.0007)	0.999 (0.997–1.0003)	0.12	0.004 (.0004)	1.004 (1.003–1.005)	< 0.001
Minimum daily minimum temperature (°C)	0.003 (0.0006)	1.003 (1.002–1.004)	< 0.001	0.002 (0.0003)	1.002 (1.002–1.003)	< 0.001

Multiple temperature variables were significant predictors and were highly colinear with other climate variables. Of note, only minimum daily maximum temperature and minimum daily minimum temperature had significant positive associations with both outcomes. These two variables were also the least colinear with maximum daily specific humidity. Minimum daily minimum temperature was chosen for inclusion in the multivariate analysis due to its statistical significance, effect size, and better model fit when compared with minimum daily maximum temperature.

### Multivariable analysis

Results of the multivariable analysis are described in Table 4. For SSI during surgical admission, minimum daily temperature was a significant predictor with every 5 °C increase resulting in a 2% increase in the odds of surgical site infection. Maximum daily specific humidity was also a significant predictor, however upon Holm’s correction specific humidity was no longer significant for SSI during admission. Maximum daily precipitation was not a significant predictor for SSI during admission.

**Table 4:** Multivariable multinomial analysis coefficients and odds ratios for predictors of surgical site infection. Procedure categories (n = 15) and MSA (n = 393) were included in analysis but omitted from the table. Years are measured from 2007 up to 2014. Adjusted p-values were derived using Holm’s method.

	SSI during admission				SSI after discharge			
	B (SE)	OR (95% CI)	p	Adjusted p	B (SE)	OR (95% CI)	p	Adjusted p
Sex (1 = female)	− 0.14 (0.14)	0.87 (0.84–0.90)	< 0.001	< 0.001	0.032 (0.008)	1.03 (1.01–1.05)	< 0.001	< 0.001
Medicare	− 0.4 (0.018)	0.67 (0.64–0.69)	< 0.001	< 0.001	− 0.31 (0.01)	0.73 (0.72–0.75)	< 0.001	< 0.001
Inpatient	2.98 (0.031)	19.7 (18.5–20.9)	< 0.001	< 0.001	0.54 (0.009)	1.72 (1.68–1.75)	< 0.001	< 0.001
Charlson comorbidity index	0.054 (0.002)	1.06 (1.05–1.06)	< 0.001	< 0.001	0.068 (0.001)	1.07 (1.07–1.07)	< 0.001	< 0.001
Length of stay	0.028 (0.0004)	1.03(1.03–1.03)	< 0.001	< 0.001	0.015 (0.0004)	1.02 (1.01–1.02)	< 0.001	< 0.001
Years	− 0.039 (0.003)	0.96 (0.96–0.97)	< 0.001	< 0.001	− 0.029 (0.002)	0.97 (0.97–0.97)	< 0.001	< 0.001
Maximum daily precipitation (cm/day)	0.002 (0.004)	1.002 (0.99–1.01)	0.65	0.065	0.009 (0.002)	1.009 (1.006–1.013)	< 0.001	< 0.001
Maximum daily specific humidity (g/kg)	− 0.008 (0.004)	0.99 (0.99–1.0)	0.037	0.07	0.026 (0.002)	1.03 (1.02–1.03)	< 0.001	< 0.001
Minimum daily minimum temperature (°C)	0.0042 (0.0015)	1.004 (1.001–1.007)	0.004	0.01	− 0.005 (0.0007)	0.995 (0.99–1.00)	< 0.001	< 0.001

Maximum daily precipitation, maximum daily specific humidity, and minimum daily minimum temperature were all significant predictors of SSI diagnosed on follow-up after Holm’s correction. Every 10 cm increase of maximum daily precipitation resulted in a 1.09 odds increase in SSI after discharge, while every g/kg unit increase in specific humidity resulted in a 1.03 odds increase in SSI risk after discharge. Of note, minimum temperature was associated with decreased odds of SSI after discharge (OR: 0.995, 95% CI 0.993–0.996). When running an identical model with specific humidity omitted, minimum daily minimum temperature was positively associated with SSI after discharge (OR: = 1.004, 95% CI 1.003–1.004). As maximum specific humidity is a function of temperature and thus partly colinear, this suggests that much of the effect of temperature on SSI after discharge is driven by an increase in specific humidity.

### Surgical site infection seasonality

**Figure 1:**
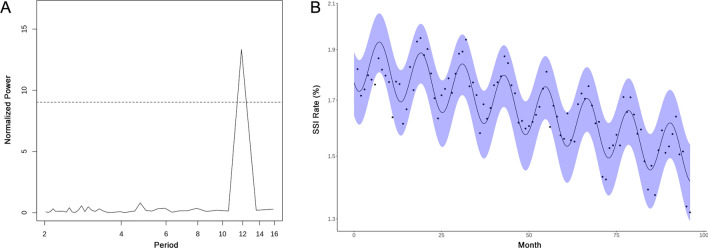
(**A**) Lomb-Scargle periodogram showing significant ~ 12 month periodicity for surgical site infections across the continental United States. Points above the dotted line show significance of p < 0.05. (**B**) Non-linear regression modeling of SSI seasonality. The x-axis represents how many months have passed since January 2007. The shaded blue area represents the model 95% CI.

Lomb-Scargle analysis showed a significant SSI periodicity of ~ 12 months (p < 0.001) (Fig. 1A). Regression of Eq. (1) showed strength of seasonality ($\epsilon$) was statistically significant and > 0, further supporting seasonality of SSI occurrence. In subgroup analysis of 5° latitude bands ranging from 25° to 50° N, this periodicity remained significant at ~ 12 months except for 25°–30° N, where there was no significant periodicity. SSI incidence peaked for procedures performed in July (p < 0.001), with an overall decreasing incidence of SSI of 0.04% (p < 0.001) per year from January 1, 2007 to December 31, 2014. Figure 1B shows the best-fit model for Eq. (1). There was an overall relative 18.1% (p < 0.001) increase in SSI rate between December, the month with the lowest SSI rate, and July across all observed years (Fig. 1B).

### Regional susceptibility analysis

**Figure 2:**
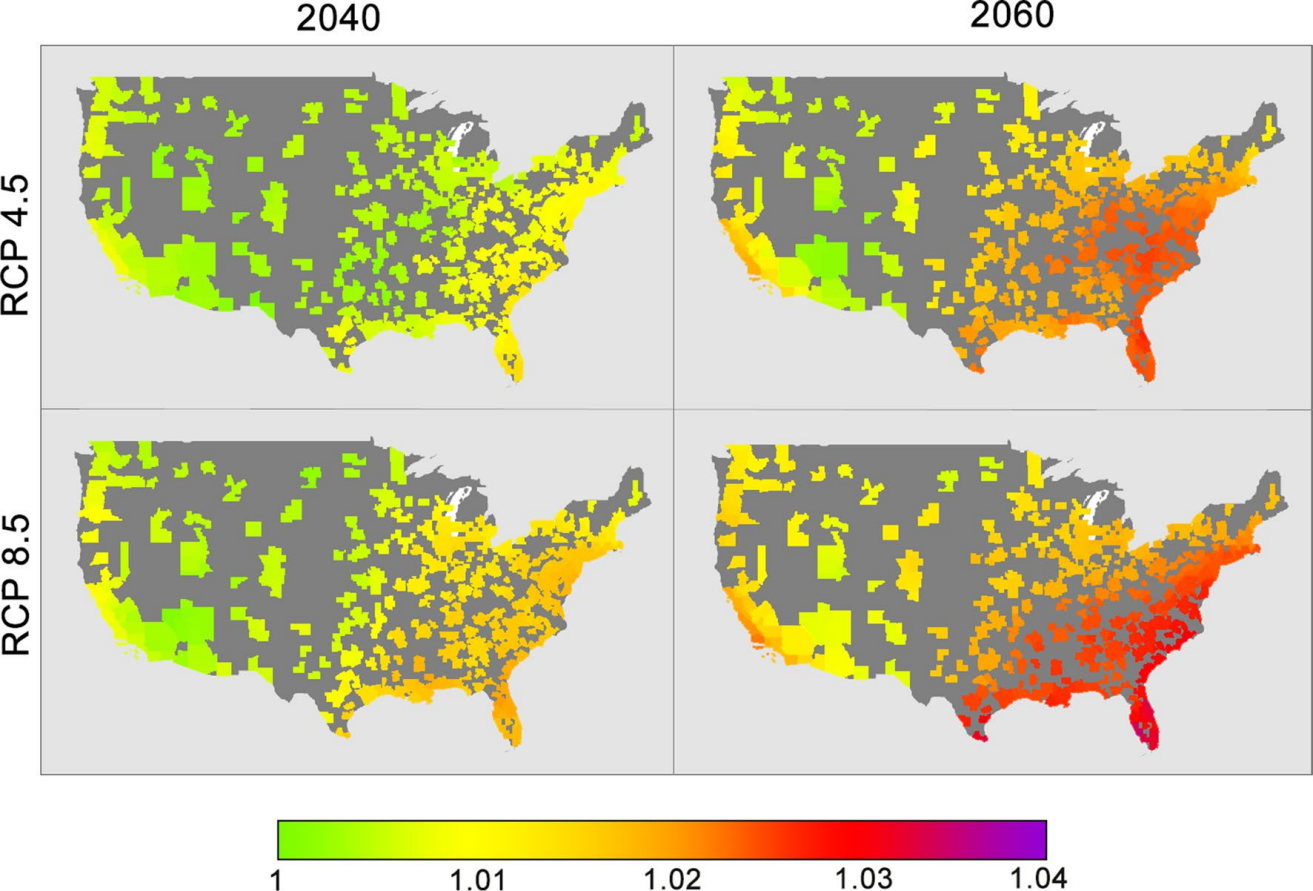
Odds ratios for surgical site infection across all 393 MSAs compared to 2010, with each figure representing a unique combination of Representative Concentration Pathway (RCP) scenarios and year of prediction. Odds ratios were calculated using CMIP5 predictions of precipitation, specific humidity, and temperature multiplied by the multivariable multinomial logistic model coefficients for SSI post-discharge.

Figure 2 shows the predicted odds ratio increase in SSI across different greenhouse gas scenarios and years. Across all MSAs, mean odds ratios for RCP 4.5 are 1.01 and 1.02 for year 2040 and 2060, respectively. For RCP 8.5, mean odds ratios are 1.01 and 1.02 for year 2040 and 2060, respectively. Supplemental Table 4 shows mean odds ratios for census regions and divisions as defined by the U.S. Census Bureau. In both intermediate and worst-case emission scenarios, the Southeast region of the United States is predicted to have the highest increase in climate-related surgical site infection risk with an odds ratio of 1.03. In particular, the South Atlantic division, composed of the states of Florida, Georgia, South Carolina, North Carolina, Virginia, West Virginia, Maryland, Delaware, and the city of Washington D.C. will see an odds increase of ~ 1.03 by 2060 in the worst-case emission scenario.

## Discussion

To our knowledge, this is the first study investigating impact of temperature, humidity, precipitation, and seasonality on SSI rates for all NHSN monitored surgical procedures during both hospital admission and post-discharge using a representative dataset of the continental United States. Previous studies have investigated the effects of meteorological conditions and seasonality on surgical site infection, however many of these studies are limited by geographic region, a small subset of surgical procedures, or poor correlation between SSI occurrence and procedure date^18–22^. The most comprehensive prior study by Anthony et al. found a significant effect of seasonality and temperature on surgical site infection across the National Inpatient Sample^22^. However, this study is limited by weather variables correlated to date of SSI admission and not date of procedure, unlinked SSI incidences to procedure type or date, and a small selection of NHSN surgical procedures used to calculate SSI incidence. Nevertheless, our findings are consistent with previous studies, with peak incidence occurring in July and nadir occurring in December. Of note, there was an annual decrease of SSI incidence of 0.04% per year, suggesting that current SSI prevention measures may be decreasing SSI incidence overall, reporting of SSIs may be decreasing or both.

For SSI during surgical admission, only minimum daily minimum temperature was found to be predictive of SSI after Holms’ correction, with every 5 °C increase in temperature correlating with a 2% increase in odds of SSI. While specific humidity was not significant after Holms’ correction, it is possible that our 15-day lead-time did not accurately capture the impact of specific humidity on SSI during admission. Previous studies have found an association between humidity and temperature with other hospital-acquired infections. For example, a retrospective study looking at skin and soft tissue infection (SSTI) incidence in adolescent Medicaid patients saw an increase in SSTI incidence with increases in mean temperature and specific humidity^11^. A prospective cohort study looking at MRSA and vancomycin-resistant enterococci (VRE) colonization in 20 ICUs in the United States found a 10% increase in relative humidity led to a 9% increase in MRSA and VRE prevalence rate^10^. Further studies into lead-time effect of temperature and humidity prior to surgical admission may better describe weather risk-windows and help guide preventative strategies for in-hospital SSI.

In contrast with SSI during surgical admission, daily maximum precipitation, daily maximum specific humidity, and minimum daily minimum temperature were all found to be significant predictors of SSI post-discharge. Overall effect size of climate variables was greater for development of SSI after discharge than while hospitalized. While precipitation was found to be a significant predictor, its effect size is minimal compared to that of temperature and humidity, and the amount of precipitation change required for significant change in SSI rates is substantial. However, future investigations using more sensitive metrics of precipitation centered around occurrence of SSI event may elucidate larger effect sizes. Of note, minimum temperature was negatively associated with SSI post-discharge, however when a similar model was run without specific humidity, the relationship between minimum daily minimum temperature and SSI rate was positively associated. This inversion of coefficients is likely a result of the collinearity existing between specific humidity and temperature. While the specifics of the mathematical relationship between specific humidity and temperature are detailed elsewhere, in brief at 100% relative humidity, every 1–2 °C increase in temperature results in a 1 g/kg increase in specific humidity depending on atmospheric temperature^45^. As the effect size of a unit g/kg increase in specific humidity on SSI is roughly 6 times greater than a 1 °C increase in temperature, the overall effect of temperature on SSI development is positive for high relative humidity. This distinction between effects of humidity and temperature is important as areas with high heat, humidity, and precipitation will likely see marked increased risk of SSI relative to other regions which may see equivalent increases in temperature without the same change in atmospheric water content.

This interdependence of climate variables has important implications when planning for future best case-worst case scenarios at the locoregional level. Using existing climate predictive models, we have shown an increase in climate-related SSI after discharge across the United States for both intermediate and worst-case emission scenarios—however the Southeast region appears particularly vulnerable. While the odds increase by 2040 represents only a 1–2% increase in SSI, the cumulative effect of an additional 1000–1500 SSIs every year across decades of climate change is substantial, particularly when considering the individual implications and cost to the healthcare system. By 2060 and in the highest emission scenario (RCP 8.5), there could be an additional 3% SSI annual increase in the most vulnerable regions. While described as the “worst-case” scenario, recent literature suggests that we are currently on-track with RCP 8.5 emissions^46^.

While exact mechanisms behind climate-related SSI remain unknown, previous research suggests increased skin flora in warm and humid conditions may contribute. Bacteria causing SSIs commonly originate from the patient’s own microbial flora, and high-temperature high-humidity conditions have been shown to increase bacterial skin colonization in certain body regions^47,48^. Methicillin-resistant *Staphylococcus aureus* colonization of the nares has long been associated with SSI and is shown to increase during the summer^49^. *Enterobacter aerogenes* has been found to grow best in high-temperature high-humidity conditions on a variety of surfaces, likely due to decreased desiccation stress^9^. While increased skin colonization during hot and humid conditions likely contributes to increased SSI incidence, other mechanisms may exist and warrant further investigation.

Our study has several important limitations. First, weather data was averaged across entire surveillance periods. Patients could have developed SSI before the surveillance window ended, potentially reducing the effect size of weather variables on SSI. Second, Marketscan^®^ does not include key demographic data such as race and income, which have been shown to be contributors to SSI in certain surgical procedures^9,35^. Populations not represented in this study include the uninsured, those on Medicaid, and those who live outside an MSA. These vulnerable populations likely represent an at-risk group of climate-related SSI potentially leading to underestimation of the future risk by our models^9^. Third, procedure characteristics such as wound class and procedure length, which are also known risk factors for SSI, were also unavailable in the dataset. Fourth, our study did not include encounters where surgeries from multiple procedure category groups occurred. As these were likely larger operations that require longer lengths of stay, it is likely that SSI in these groups would be even higher than those reported in our study, suggesting our predictions are underestimates. Fifth, we grouped NHSN procedure categories into larger procedure category groups to help prevent overfitting of the model, however NHSN specific subgroup analysis could help elucidate which procedure groups in particular are at risk for climate-related SSI and receive appropriate adjustment in NHSN standardized infection ratio calculations. Sixth, infections were defined by ICD9 code, and so are susceptible to coding error. However, a meta-analysis showed ICD9 codes 998.51 and 998.59 have a sensitivity and specificity of 84.1% and 97.1% respectively^50^. Future studies that include bacterial microbial data and further subgrouping of SSI into superficial, deep, or organ space categories could help illuminate which microbes and infection types are most associated with climate-related SSI. Seventh, this study relied on healthcare administrative data which depends on the veracity of the coding, and does not include independently verified clinical outcomes. Eighth, it is possible that future surgical technique will be associated with lower risk of SSIs—this potential for improved technology was not accounted for in our models which could lead to over-representation of future risk. Ninth, the CMIP5 climate prediction models did not include error ranges, thus it is difficult to estimate what the upper and lower bounds of future SSI risk may be. Tenth, seasonality, temperature, humidity, and precipitation are interrelated. We elected to demonstrate seasonal impact on SSI incidence using the Lomb-Scargle periodogram. It is possible that some of the greater future risk in the southeastern US could be attributable to decreased seasonal variation, rather than humidity or temperature changes alone. Future studies could compare statistical methods to account for seasonality to further elucidate the inter-relatedness of these climactic variable. Finally, we were not able to account for the humidity and temperature experienced while a patient was hospitalized or other important in-hospitalization variables known to contribute to development of SSI. For this study we assumed that variability in these practices is homogenous across the United States.

In conclusion, we were able to describe a modest effect of meteorological conditions and season on SSI across NHSN monitored procedures occurring within the continental United States. Using climate modeling scenarios, we identified the Southeast region of the United States as particularly vulnerable to future climate-related SSI. Further investigations into the mechanisms of climate-related SSI, the lead-lag time between weather conditions and development of SSI, and inclusion of more vulnerable groups both domestically and abroad are warranted. These studies could help develop prevention strategies and further identify at risk groups of climate-related SSI.

## Supplementary Information

Supplementary Table 1.

Supplementary Table 2.

Supplementary Table 3.

Supplementary Table 4.

## Data Availability

The data that support the findings of this study are available from Stanford Center for Population Health Sciences Data Core but restrictions apply to the availability of these data, which were used under license for the current study, and so are not publicly available. Data are however available from the corresponding author upon reasonable request and with permission of Stanford Center for Population Health Sciences Data Core.
