# Supplementary material for: Effect of climate on surgical site infections and anticipated increases in the United States
Source: Sci Rep. 2022 Nov 16;12:19698. doi: 10.1038/s41598-022-24255-w (PMC9668825; doi:10.1038/s41598-022-24255-w)
Supplement: Supplementary file 1 — Supplementary Table 1. [file 41598_2022_24255_MOESM1_ESM.docx]

| **Procedure Code Category** | **ICD-9 and CPT Codes** |
| --- | --- |
| Abdominal aortic aneurysm repair | 34830, 34831, 34832, 35081, 35082, 35091, 35092, 35102, 35103, 3834, 3844, 3864, 3884 |
| Limb amputation | 23900, 23920, 24900, 24920, 24930, 24931, 25900, 25905, 25909, 25920, 25922, 25924, 25927, 25929, 25931, 26235, 26236, 26551, 26910, 26951, 26952, 27290, 27295, 27590, 27591, 27592, 27598, 27880, 27881, 27882, 27884, 27886, 27888, 27889, 28124, 28126, 28160, 28800, 28805, 28810, 28820, 28825, 8400, 8401, 8402, 8403, 8404, 8405, 8406, 8407, 8408, 8409, 8410, 8411, 8412, 8413, 8414, 8415, 8416, 8417, 8418, 8419 |
| Appendix surgery | 44900, 44950, 44955, 44960, 44970, 44979, 4701, 4709, 4711, 4719, 4791, 4799 |
| Shunt for dialysis | 36800, 36810, 36815, 36818, 36819, 36820, 36821, 36825, 36830, 36832, 36833, 36838, 3942 |
| Bile duct, liver, or pancreatic surgery | 0585T, 0586T, 47010, 47015, 47100, 47120, 47122, 47125, 47130, 47140, 47141, 47142, 47300, 47350, 47360, 47361, 47362, 47370, 47371, 47379, 47380, 47381, 47400, 47420, 47425, 47460, 47700, 47701, 47711, 47712, 47715, 47760, 47765, 47780, 47785, 47800, 47802, 47900, 48000, 48001, 48020, 48100, 48105, 48120, 48140, 48145, 48146, 48148, 48150, 48152, 48153, 48154, 48155, 48160, 48500, 48510, 48520, 48540, 48545, 48548, 500, 5011, 5012, 5014, 5021, 5022, 5023, 5025, 5026, 5029, 503, 504, 5061, 5069, 5091, 5099, 5113, 5114, 5135, 5136, 5137, 5139, 5141, 5142, 5149, 5151, 5159, 5161, 5162, 5163, 5164, 5169, 5171, 5172, 5179, 5181, 5182, 5183, 5184, 5185, 5187, 5188, 5189, 5198, 5199, 5201, 5209, 5211, 5212, 5214, 5221, 5222, 5251, 5252, 5253, 5259, 526, 527, 5280, 5281, 5282, 5283, 5284, 5285, 5286, 5292, 5293, 5294, 5295, 5296, 5298, 5299 |
| Breast surgery | 11970, 19101, 19105, 19110, 19112, 19120, 19125, 19126, 19300, 19301, 19302, 19303, 19305, 19306, 19307, 19316, 19318, 19324, 19325, 19328, 19330, 19340, 19342, 19350, 19355, 19357, 19361, 19364, 19366, 19367, 19368, 19369, 19370, 19371, 19380, 8377, 850, 8512, 8520, 8521, 8522, 8523, 8524, 8525, 8531, 8532, 8533, 8541, 8542, 8550, 8553, 8554, 856, 8570, 8571, 8572, 8573, 8574, 8575, 8576, 8579, 8589, 8593, 8594, 8595, 8596 |
| Cardiac surgery | 0051T, 0052T, 0053T, 32658, 32659, 32661, 33020, 33025, 33030, 33031, 33050, 33120, 33130, 33250, 33251, 33254, 33255, 33256, 33257, 33258, 33259, 33261, 33265, 33266, 33300, 33305, 33310, 33315, 33365, 33366, 33390, 33391, 33404, 33405, 33406, 33410, 33411, 33412, 33413, 33414, 33415, 33416, 33417, 33420, 33422, 33425, 33426, 33427, 33430, 33440, 33460, 33463, 33464, 33465, 33468, 33470, 33471, 33474, 33475, 33476, 33478, 33496, 33542, 33545, 33548, 33600, 33602, 33608, 33610, 33611, 33612, 33615, 33617, 33619, 33641, 33645, 33647, 33660, 33665, 33670, 33675, 33676, 33677, 33681, 33684, 33688, 33692, 33694, 33697, 33702, 33710, 33720, 33722, 33732, 33735, 33736, 33737, 33770, 33774, 33776, 33780, 33782, 33783, 33786, 33813, 33814, 33920, 33975, 33976, 33977, 33978, 33979, 33980, 56, 3500, 3501, 3502, 3503, 3504, 3510, 3511, 3512, 3513, 3514, 3520, 3521, 3522, 3523, 3524, 3525, 3526, 3527, 3528, 3531, 3532, 3533, 3550, 3551, 3552, 3553, 3554, 3555, 3560, 3561, 3562, 3563, 3570, 3571, 3572, 3573, 3581, 3582, 3583, 3591, 3592, 3593, 3594, 3595, 3596, 3597, 3598, 3599, 362, 3631, 3632, 3633, 3699, 370, 3710, 3711, 3712, 3724, 3725, 3731, 3733, 3735, 3736, 3737, 3741, 3749, 3752, 3753, 3754, 3755, 3760, 3762, 3763, 3764, 3765, 3766, 3768, 3790, 3799, 3826, 3959 |
| Coronary artery bypass graft with both chest and donor site incisions or chest incision only | 33510, 33511, 33512, 33513, 33514, 33516, 3610, 3611, 3612, 3613, 3614, 3615, 3616, 3617, 3619, 33533, 33534, 33535, 33536, 3611, 3612, 3613, 3614, 3615, 3616, 3619, 3639 |
| Carotid endarterectomy | 35301, 35390, 3802, 3812 |
| Gallbladder surgery | 47480, 47562, 47563, 47564, 47570, 47600, 47605, 47610, 47612, 47620, 47720, 47721, 47740, 47741, 5101, 5102, 5103, 5104, 5112, 5121, 5122, 5123, 5124, 5131, 5132, 5133, 5134, 5191, 5192, 5193, 5194, 5195 |
| Colon surgery | 44025, 44110, 44111, 44137, 44140, 44141, 44143, 44144, 44145, 44146, 44147, 44150, 44151, 44155, 44156, 44157, 44158, 44160, 44188, 44204, 44205, 44206, 44207, 44208, 44210, 44211, 44212, 44213, 44227, 44320, 44322, 44340, 44345, 44346, 44604, 44605, 44620, 44625, 44626, 1731, 1732, 1733, 1734, 1735, 1736, 1739, 4438, 4439, 4502, 4503, 4514, 4515, 4525, 4526, 4527, 4533, 4534, 4541, 4543, 4549, 4562, 4571, 4572, 4573, 4574, 4575, 4576, 4579, 4581, 4582, 4583, 4593, 4594, 4603, 4604, 4610, 4611, 4613, 4660, 4664, 4671, 4672, 4673, 4674, 4675, 4676, 4679, 4680, 4682, 4687, 4691, 4692, 4693, 4694 |
| Craniotomy | 61105, 61107, 61108, 61120, 61140, 61150, 61151, 61154, 61156, 61210, 61250, 61253, 61304, 61305, 61312, 61313, 61314, 61315, 61320, 61321, 61322, 61323, 61330, 61333, 61340, 61343, 61345, 61458, 61460, 61510, 61512, 61514, 61516, 61518, 61519, 61520, 61521, 61522, 61524, 61526, 61530, 61531, 61533, 61534, 61535, 61536, 61537, 61538, 61539, 61540, 61541, 61543, 61544, 61545, 61546, 61548, 61566, 61567, 61570, 61571, 61575, 61576, 61580, 61581, 61582, 61583, 61584, 61585, 61586, 61590, 61591, 61592, 61595, 61598, 61600, 61601, 61605, 61606, 61607, 61608, 61615, 61616, 61618, 61619, 61680, 61682, 61684, 61686, 61690, 61692, 61697, 61698, 61700, 61702, 61703, 61705, 61708, 61710, 61711, 61720, 61735, 61750, 61751, 61760, 61770, 61850, 61860, 61863, 61867, 61870, 61880, 62000, 62005, 62010, 62100, 62120, 62121, 62161, 62163, 62164, 62165, 101, 102, 109, 111, 112, 113, 114, 118, 120, 121, 122, 123, 124, 125, 126, 127, 128, 131, 132, 139, 141, 142, 151, 152, 153, 159, 211, 212, 213, 214, 221, 222, 233, 234, 291, 292, 293, 296, 301, 713, 714, 715, 717, 751, 752, 753, 754, 759, 761, 762, 763, 764, 765, 768, 769, 771, 772, 779, 1754, 3801, 3811, 3821, 3831, 3841, 3861, 3881, 3898, 3928, 3929, 3950, 3951, 3952, 3956, 3957, 3958, 3972, 3974, 3975, 3976, 3979, 3990, 45, 46, 47, 48, 55, 62, 65 |
| Cesarean section | 59100, 59510, 59514, 59515, 59618, 59620, 59622, 59857, 680, 740, 741, 742, 743, 744, 7491, 7499 |
| Spinal fusion | 22532, 22533, 22548, 22551, 22554, 22556, 22558, 22586, 22590, 22595, 22600, 22610, 22612, 22630, 22633, 22800, 22802, 22804, 22808, 22810, 22812, 27280, 8100, 8101, 8102, 8103, 8104, 8105, 8106, 8107, 8108, 8162, 8163, 8164, 8451 |
| Open reduction of fracture | 23615, 23616, 23630, 23670, 23680, 24515, 24516, 24545, 24546, 24575, 24579, 24586, 24587, 24635, 24665, 24666, 24685, 25337, 25515, 25525, 25526, 25545, 25574, 25575, 25607, 25608, 25609, 25652, 27177, 27178, 27179, 27181, 27244, 27245, 27248, 27254, 27269, 27506, 27507, 27511, 27513, 27514, 27519, 27535, 27536, 27540, 27758, 27759, 27766, 27769, 27784, 27792, 27814, 27822, 27823, 27826, 27827, 27828, 27829, 7812, 7813, 7815, 7817, 785, 7852, 7853, 7921, 7922, 7925, 7926, 7931, 7932, 7935, 7936, 8471, 8472, 8473 |
| Gastric surgery | 43117, 43118, 43121, 43122, 43286, 43287, 43288, 43320, 43360, 43500, 43501, 43502, 43520, 43605, 43610, 43611, 43620, 43621, 43622, 43631, 43632, 43633, 43634, 43640, 43641, 43644, 43645, 43770, 43771, 43772, 43773, 43774, 43775, 43800, 43810, 43820, 43825, 43840, 43842, 43843, 43845, 43846, 43847, 43848, 43850, 43855, 43860, 43865, 43870, 43880, 43886, 43887, 43888, 4209, 4224, 4225, 4232, 4239, 4252, 430, 4311, 4319, 433, 4341, 4342, 4349, 435, 436, 437, 4381, 4382, 4389, 4391, 4399, 4400, 4401, 4402, 4403, 4414, 4415, 4421, 4422, 4429, 4431, 4440, 4441, 4442, 445, 4464, 4465, 4466, 4467, 4468, 4469, 4495, 4496, 4497, 4499 |
| Herniorrhaphy | 11008, 49491, 49492, 49495, 49496, 49500, 49501, 49505, 49507, 49520, 49521, 49525, 49550, 49553, 49555, 49557, 49560, 49561, 49565, 49566, 49570, 49572, 49580, 49582, 49585, 49587, 49590, 49600, 49605, 49606, 49610, 49611, 49650, 49651, 49652, 49653, 49654, 49655, 49656, 49657, 49659, 1711, 1712, 1713, 1721, 1722, 1723, 1724, 5300, 5301, 5302, 5303, 5304, 5305, 5310, 5311, 5312, 5313, 5314, 5315, 5316, 5317, 5321, 5329, 5331, 5339, 5341, 5342, 5343, 5349, 5351, 5359, 5361, 5362, 5363, 5369, 5471, 5472 |
| Hip prosthesis | 27125, 27130, 27132, 27134, 27137, 27138, 27236, 74, 75, 76, 77, 85, 86, 87, 8005, 8015, 8016, 8151, 8152, 8153, 8457 |
| Heart transplant | 33935, 33945, 3751 |
| Abdominal hysterectomy | 58150, 58152, 58180, 58200, 58210, 58240, 58541, 58542, 58543, 58544, 58548, 58550, 58552, 58553, 58554, 58570, 58571, 58572, 58573, 58575, 58951, 58953, 58954, 58956, 59525, 6831, 6839, 6841, 6849, 6851, 6861, 6869, 6871, 6879, 689 |
| Knee prosthesis | 27438, 27440, 27441, 27442, 27443, 27445, 27446, 27447, 27486, 27487, 8006, 8154, 8155 |
| Kidney transplant | 50340, 50360, 50365, 50380, 5569 |
| Laminectomy | 0202T, 0219T, 0220T, 0221T, 22220, 22222, 22224, 22856, 22857, 22861, 22862, 22867, 62287, 62351, 62380, 63001, 63003, 63005, 63011, 63012, 63015, 63016, 63017, 63020, 63030, 63035, 63040, 63042, 63045, 63046, 63047, 63048, 63050, 63051, 63055, 63056, 63064, 63075, 63077, 63081, 63082, 63085, 63086, 63087, 63088, 63090, 63091, 63101, 63102, 63103, 63170, 63172, 63173, 63180, 63182, 63185, 63190, 63191, 63194, 63195, 63196, 63197, 63198, 63199, 63200, 63250, 63251, 63252, 63265, 63266, 63267, 63268, 63270, 63271, 63272, 63273, 63275, 63276, 63277, 63278, 63280, 63281, 63282, 63283, 63285, 63286, 63287, 63290, 63709, 63740, 309, 321, 329, 331, 332, 339, 34, 351, 359, 36, 371, 372, 397, 399, 449, 352, 353, 379 |
| Liver transplant | 47135, 5051, 5059 |
| Neck surgery | 31300, 31360, 31365, 31367, 31368, 31370, 31375, 31380, 31382, 31390, 31395, 31400, 31420, 31551, 31552, 31553, 31554, 31560, 31561, 31580, 31584, 31587, 31590, 31591, 31592, 31599, 38308, 38542, 38720, 38724, 41135, 41140, 41145, 41155, 42420, 42425, 42426, 69150, 69155, 303, 304, 3009, 3021, 3022, 3029, 311, 3121, 3129, 313, 3143, 3144, 3145, 315, 3179, 3198, 4040, 4041, 4042 |
| Kidney surgery | 50010, 50020, 50040, 50045, 50060, 50065, 50070, 50075, 50120, 50125, 50130, 50135, 50205, 50220, 50225, 50230, 50234, 50236, 50240, 50250, 50280, 50290, 50320, 50400, 50405, 50541, 50542, 50543, 50545, 50546, 50547, 50548, 50549, 5502, 5503, 5504, 5511, 5512, 5523, 5524, 5532, 5534, 5539, 554, 5551, 5552, 5553, 5554, 5586, 5589, 5591, 5592, 5673, 5921, 5991, 5995 |
| Ovarian surgery | 58660, 58661, 58662, 58679, 58720, 58740, 58800, 58805, 58820, 58822, 58825, 58900, 58920, 58925, 58940, 58943, 58950, 58952, 58970, 6501, 6509, 6512, 6513, 6522, 6524, 6525, 6529, 6531, 6539, 6541, 6549, 6551, 6552, 6553, 6554, 6561, 6562, 6563, 6564, 6571, 6572, 6573, 6574, 6575, 6576, 6579, 6581, 6589, 6592, 6599 |
| Prostate surgery | 55705, 55810, 55812, 55815, 55821, 55831, 55840, 55842, 55845, 55866, 600, 6011, 6012, 6015, 603, 604, 605, 6061, 6062, 6069, 6081, 6082, 6091 |
| Peripheral vascular bypass surgery | 33889, 35501, 35506, 35508, 35509, 35510, 35511, 35512, 35515, 35516, 35518, 35521, 35522, 35523, 35525, 35533, 35556, 35558, 35566, 35570, 35571, 35583, 35585, 35587, 35616, 35621, 35623, 35650, 35654, 35656, 35661, 35666, 35671, 35686, 3582, 390, 391, 3921, 3922, 3923, 3924, 3925, 3926, 3927 |
| Rectal surgery | 45110, 45111, 45112, 45113, 45114, 45116, 45119, 45120, 45121, 45126, 45395, 45397, 45562, 45563, 57307, 4592, 4595, 480, 481, 4824, 4825, 4826, 4840, 4841, 4842, 4843, 4849, 4850, 4851, 4852, 4859, 4861, 4862, 4863, 4864, 4865, 4869, 4875, 4879, 496, 4994 |
| Small bowel surgery | 43496, 44010, 44020, 44021, 44120, 44125, 44126, 44127, 44130, 44186, 44187, 44202, 44300, 44310, 44312, 44314, 44316, 44602, 44603, 44615, 44640, 44650, 44800, 45136, 4432, 4438, 4439, 4500, 4501, 4502, 4502, 4503, 4514, 4515, 4530, 4531, 4532, 4533, 4534, 4561, 4562, 4563, 4591, 4601, 4602, 4620, 4621, 4622, 4623, 4639, 4660, 4661, 4662, 4679, 4680, 4681, 4687, 9751, 9752, 9759 |
| Spleen surgery | 38100, 38101, 38102, 38115, 38120, 411, 412, 4133, 4142, 4143, 415, 4195 |
| Thoracic surgery | 20101, 21603, 31770, 31775, 32096, 32097, 32100, 32110, 32120, 32124, 32140, 32141, 32151, 32200, 32215, 32220, 32225, 32310, 32320, 32440, 32442, 32445, 32480, 32482, 32484, 32486, 32488, 32491, 32501, 32503, 32504, 32505, 32506, 32507, 32540, 32553, 32607, 32608, 32609, 32651, 32652, 32655, 32662, 32663, 32666, 32667, 32668, 32669, 32670, 32671, 32672, 32800, 32815, 32905, 32906, 32940, 32960, 39000, 39010, 39200, 39220, 39501, 39545, 39560, 39561, 64746, 42, 525, 716, 780, 781, 782, 791, 792, 793, 794, 798, 3201, 3209, 321, 3220, 3221, 3222, 3223, 3224, 3225, 3226, 3228, 3229, 3230, 3239, 3241, 3249, 3250, 3259, 330, 331, 3320, 3324, 3325, 3326, 3328, 3334, 3348, 3349, 3392, 3393, 3401, 3402, 3403, 3405, 3406, 3409, 341, 3420, 3421, 3422, 3424, 3426, 3427, 343, 344, 3451, 3452, 3459, 346, 3479, 3481, 3484, 3485, 3489, 3493, 3499, 3998, 400, 4011, 4069, 409, 5371, 5372, 5375, 8382 |
| Thyroid and/or parathyroid surgery | 60000, 60200, 60210, 60212, 60220, 60225, 60240, 60252, 60254, 60260, 60270, 60271, 60280, 60281, 60500, 60502, 60505, 60512, 601, 609, 611, 612, 613, 619, 62, 631, 639, 64, 650, 651, 652, 67, 681, 689, 691, 693, 694, 695, 698, 699 |
| Vaginal hysterectomy | 51925, 58260, 58262, 58263, 58267, 58270, 58275, 58280, 58285, 58290, 58291, 58292, 58293, 58294, 6859 |
| Ventricular shunt | 62160, 62180, 62190, 62192, 62194, 62201, 62220, 62223, 62225, 62230, 62252, 62256, 62258, 231, 232, 235, 239, 242, 243, 8609 |
| Exploratory laparotomy | 20102, 35840, 39503, 39540, 39541, 43332, 43333, 44005, 44180, 44700, 44820, 44850, 49000, 49002, 49010, 49013, 49014, 49020, 49040, 49060, 49203, 49204, 49205, 49215, 49220, 49250, 49255, 49320, 49321, 49324, 49325, 49326, 49402, 49412, 49419, 49421, 49425, 49426, 49900, 49905, 49906, 58960, 3191, 4019, 4139, 4221, 4229, 4403, 4411, 4429, 4449, 4469, 4511, 4521, 4821, 4881, 4882, 4891, 4979, 4991, 5119, 5219, 540, 5411, 5412, 5419, 5421, 5422, 5423, 5424, 5429, 543, 544, 5451, 5459, 5464, 5473, 5474, 5475, 5491, 5492, 5493, 5494, 5495, 5499, 5501, 5521, 562, 580, 5900, 5909, 5929, 8628, 9786 |

Supplemental Table 1. ICD-9 and CPT Codes used to filter MarketScan records. ICD-9 codes are translated from ICD-10 codes and formatted for filtering Marketscan records. Translated ICD-9 codes which occurred in multiple procedure code categories were omitted.
