# Supplementary material for: Effect of climate on surgical site infections and anticipated increases in the United States
Source: Sci Rep. 2022 Nov 16;12:19698. doi: 10.1038/s41598-022-24255-w (PMC9668825; doi:10.1038/s41598-022-24255-w)
Supplement: Supplementary file 2 — Supplementary Table 2. [file 41598_2022_24255_MOESM2_ESM.docx]

| **Models for Temperature and Precipitation** | **Models for Specific Humidity** |
| --- | --- |
| ACCESS1-0 | ACCESS1-3 |
| BCC-CSM1-1 | CanESM2 |
| BCC-CSM1-1-M | CNRM-CM5 |
| BNU-ESM | CSIRO-MK3-6-0 |
| CanESM2 | GFDL-CM3 |
| CCSM4 | GISS-E2-H-CC |
| CESM1-BGC | HadGEM2-AO |
| CESM1-CAM5 | INMCM4 |
| CMCC-CM | MIROC-ESM |
| CNRM-CM5 | MRI-CGCM3 |
| CSIRO-MK3-6-0 | NorESM1-M |
| FGOALS-g2 |  |
| FIO-ESM |  |
| GFDL-CM3 |  |
| GFDL-ESM2G |  |
| GFDL-ESM2M |  |
| GISS-E2-H-CC |  |
| GISS-E2-R |  |
| GISS-E2-R-CC |  |
| HadGEM2-AO |  |
| HadGEM2-CC |  |
| HadGEM2-ES |  |
| INMCM4 |  |
| IPSL-CM5A-LR |  |
| IPSL-CM5A-MR |  |
| IPSL-CM5B-LR |  |
| MIROC-ESM |  |
| MIROC-ESM-CHEM |  |
| MIROC5 |  |
| MPI-ESM-LR |  |
| MPI-ESM-MR |  |
| MRI-CGCM3 |  |
| NorESM1-M |  |

Supplemental Table 2. General Circulation Models (GCM) conducted under the Coupled Model Intercomparison Project Phase 5 (CMIP5) used for assessing future climate conditions and climate-related surgical site infections. Temperature and precipitation data was derived from the NASA Earth Exchange (NEX) Downscaled Climate Projections (NEX-DCP30), which is comprised of downscaled CMIP5 GCMs.
