# Supplementary material for: Effect of climate on surgical site infections and anticipated increases in the United States
Source: Sci Rep. 2022 Nov 16;12:19698. doi: 10.1038/s41598-022-24255-w (PMC9668825; doi:10.1038/s41598-022-24255-w)
Supplement: Supplementary file 3 — Supplementary Table 3. [file 41598_2022_24255_MOESM3_ESM.docx]

| **Procedure Categories** | **Number of Procedures (% total)** |
| --- | --- |
| Abdominal | 1,635,175 (21.2%) |
| C-section | 1,008,056 (13.1%) |
| Prosthesis | 870,623 (11.3%) |
| Gynecology | 814686 (10.6%) |
| Hernia | 717,522 (9.3%) |
| Breast | 598,977 (7.8%) |
| Spine | 530,975 (6.9%) |
| Fracture | 360,981 (4.7%) |
| Thoracic | 327,368 (4.2%) |
| Neuro | 205,928 (2.7%) |
| Neck | 192,635 (2.5%) |
| Urology | 150,797 (2%) |
| Vascular | 147,146 (1.9%) |
| Amputation | 132,616 (1.7%) |
| Transplant | 9,361 (0.1%) |

Supplemental Table 3. Number of procedure occurrences within each procedure category group.
