# Supplementary material for: Effect of climate on surgical site infections and anticipated increases in the United States
Source: Sci Rep. 2022 Nov 16;12:19698. doi: 10.1038/s41598-022-24255-w (PMC9668825; doi:10.1038/s41598-022-24255-w)
Supplement: Supplementary file 4 — Supplementary Table 4. [file 41598_2022_24255_MOESM4_ESM.docx]

|  | 2040 | | 2060 | |  | 2040 | | 2060 | |
| --- | --- | --- | --- | --- | --- | --- | --- | --- | --- |
| **Region** | RCP 4.5 | RCP 8.5 | RCP 4.5 | RCP 8.5 | **Division** | RCP 4.5 | RCP 8.5 | RCP 4.5 | RCP 8.5 |
| Northeast | 1.009 | 1.014 | 1.019 | 1.022 | New England | 1.009 | 1.013 | 1.017 | 1.022 |
|  |  |  |  |  | Middle Atlantic | 1.009 | 1.015 | 1.02 | 1.022 |
| South | 1.009 | 1.015 | 1.022 | 1.026 | South Atlantic | 1.01 | 1.017 | 1.024 | 1.028 |
|  |  |  |  |  | East South Central | 1.008 | 1.015 | 1.022 | 1.025 |
|  |  |  |  |  | West South Central | 1.005 | 1.011 | 1.018 | 1.023 |
| Midwest | 1.006 | 1.011 | 1.017 | 1.017 | East North Central | 1.006 | 1.012 | 1.018 | 1.018 |
|  |  |  |  |  | West North Central | 1.005 | 1.008 | 1.014 | 1.015 |
| West | 1.005 | 1.006 | 1.009 | 1.013 | Mountain | 1.003 | 1.005 | 1.006 | 1.01 |
|  |  |  |  |  | Pacific | 1.007 | 1.008 | 1.019 | 1.015 |

Supplemental Table 4. Predicted odds ratios of SSI based off CMIP5 climate projections and multivariate analysis coefficients for US Census defined regions and divisions. RCP 4.5 = intermediate emissions scenario. RCP 8.5 = worst-case emissions scenario.
